# A Case Showing a New Diagnostic Aspect of the Application of Radiofrequency Echographic Multi-Spectrometry (REMS)

**DOI:** 10.3390/diagnostics13203224

**Published:** 2023-10-17

**Authors:** Nikola Kirilov, Fabian Bischoff, Stoyanka Vladeva, Elena Bischoff

**Affiliations:** 1 Department of Orthopedics and Traumatology, Faculty of Medicine, Medical University—Pleven, 5800 Pleven, Bulgaria; 2 IPSMP Rheumatology, 6000 Stara Zagora, Bulgaria; bischoff.f@web.de; 3 Department of Health Care, Faculty of Medicine, Trakia University, 6015 Stara Zagora, Bulgaria; drvladeva@abv.bg; 4 Faculty of Public Health and Healthcare, University “Prof Dr Asen Zlatarov”—Burgas, 8010 Burgas, Bulgaria; elenabischoffmd@web.de

**Keywords:** osteoporosis, osteodensitometry, REMS, novel application, hip, fracture detection, occult fracture

## Abstract

Radiofrequency echographic multi-spectrometry (REMS) is an ultrasound technique that has been recently introduced in the medical field to detect osteoporosis and fracture risk at axial sites. The use of sonography to visualize the region of interest (ROI) of the hip neck provides the opportunity to identify occult fractures. A 91-year-old woman with persistent right leg pain was referred to rheumatologist due to a known history of arthritis and osteoporosis. She was able to walk using a crutch, although experiencing an antalgic gait. The patient had recently fallen on her right side from standing height. During the visualization of the ROI of the right femoral neck using REMS, an abrupt break of the femoral cortex suspected to be a fracture was seen; therefore, the measurement of the femoral neck was performed on the left side. The T-score had value of −2.9 SD and the fragility score was 86.7. Due to unclear signs of a fracture after an X-ray of the hip, a computed tomography (CT) exam of the hip was performed, which revealed a femoral neck fracture. Occult fractures of the femoral neck are challenging to diagnose and require numerous radiologic exams. The use of ultrasound as a method to measure bone density allows the simultaneous diagnosis of osteoporosis and detection of fractures.

**Figure 1:**
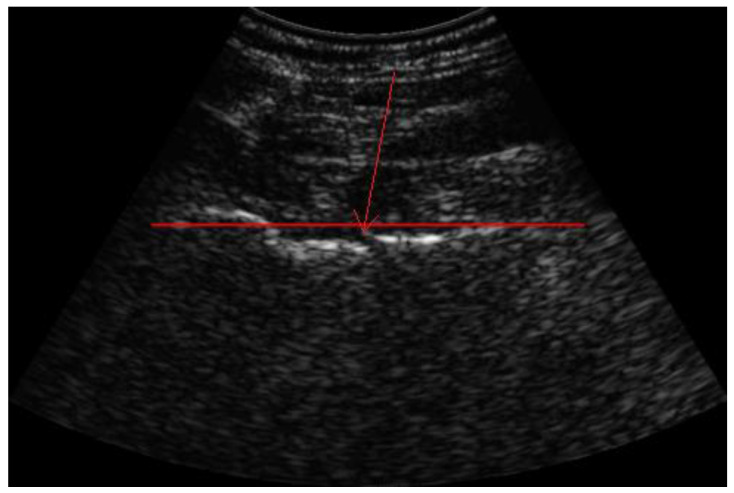
Visualization of ROI of the femoral neck using REMS for bone density measurement. Red arrow showing abrupt break of the bone cortex. In recent years, a new non-ionizing technique called radiofrequency echographic multi-spectrometry (REMS) has been introduced, which captured the interest of many medical specialists in the field of osteoporosis after single- and multi-center studies demonstrated the diagnostic accuracy of REMS compared to dual-energy X-ray absorptiometry (DEXA) [1,2,3,4]. During the evaluation with REMS, raw unfiltered native ultrasound signals, so-called radio frequency (RF) ultrasound signals, are analyzed. The spectra of the analyzed signals are compared with reference spectral models to obtain a DEXA-equivalent bone mineral density (BMD) of the lumbar spine and/or hip [5]. Based on the BMD value, subjects are classified as healthy (T-score > −1.0 SD), osteopenic (T-score between −1 and −2.5 SD) and osteoporotic (T-score of < −2.5 SD) according to the definition of the World Health Organization (WHO) [6]. The T-score represents a standard deviation of the BMD from the average BMD of the young adult reference population [7]. In addition to the T-score, microarchitectural deterioration of the bone tissue was found to be important for the diagnosis of osteoporosis due to its association with an increased risk of fractures [8]. In this context, the novel fragility score (FS) parameter, obtained during the REMS scan of lumbar spine and/or femoral regions, has been developed to estimate the ultrasound-based skeletal fragility. The FS value is acquired through comparison between the patient-specific spectral profiles with models of “fractured” and “non-fractured” subjects, which gives information about the quality of the bone microarchitecture independently of BMD [9]. A 91-year-old woman with persistent right leg pain was referred to her treating rheumatologist for examination. The clinical examination narrowed the pain down to the right hip, radiating down to the knee. The palpation of the greater trochanter was painful and there were no signs of a shortened, externally rotated or abducted leg. Straight leg raise was performed with great difficulty. She was able to walk using crutch for support, although experiencing an antalgic gait. The patient reported to have recently fallen on her right side from standing height. Due to a known history of arthritis and osteoporosis treated with bisphosphonate and denosumab since 2016, a bone density measurement was performed using REMS by a certified orthopedist. The T-score of the lumbar spine was −2.6 SD and BMD was 0.761 g/cm^2^, with a fragility score of 72. During the visualization of the region of interest (ROI) of the right femoral neck, an abrupt break of the cortex (red arrow) of the femur with a suspected fracture was seen; therefore, the measurement of the femoral neck was performed on the left side. The T-score had value of −2.9 SD and BMD was 0.527 g/cm^2^ with fragility score of 86.7.

**Figure 2:**
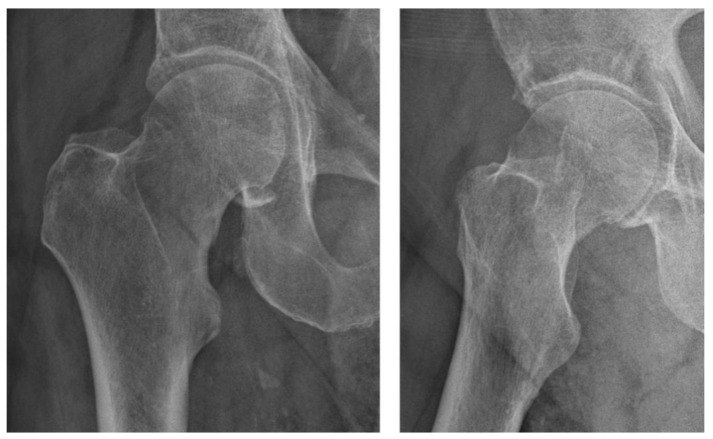
Plain radiograph of the right hip showing no clear signs of a fracture. The doctor then performed an X-ray imaging of the hip, which did not give clear signs of a fracture. Occult fractures are a type of nondisplaced fracture, which are not visible on plain radiographs or, sometimes, computed tomography (CT). This makes them prone to diagnostic errors, requiring a detailed anamnesis and clinical examination. The prevalence of such fractures is relatively high among children [10], whereas in adults the prevalence is under 10% [11,12]. An occult fracture should always be suspected when clinical symptoms and examination do not match the radiographic finding [12]. After X-ray, the next diagnostic step is the CT, which does not always exclude a false negative result. In addition to plain radiographs and CT, magnetic resonance imaging (MRI) plays an important role. Unfortunately, facilities equipped with MRI scanners are not available nationwide in all countries, and the cost–benefit has not yet been proven [13].

**Figure 3:**
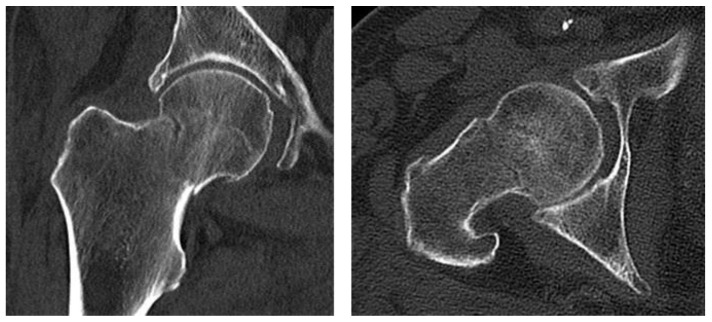
Computed tomography of the right hip reveals the femoral neck fracture. Considering the ultrasound finding and the clinical symptoms, a CT exam of the hip was performed, which revealed a femoral neck fracture. Due to the unquestionable nature of the image, we did not consider MRI for further diagnosis. The patient was then scheduled for a hemiarthroplasty of the right hip joint in the department of orthopedic surgery due to the old age of the patient. Ultrasound is not the primary diagnostic method for discovering hip fractures. Despite that, numerous studies and case reports have shown its reliability and potential in diagnosing occult femoral neck fractures where CT and X-ray fall short [14]. REMS is an accurate non-ionizing method for measuring the bone density at axial skeleton sites, and its results highly correlate to those measured using DEXA [2]. Moreover, the use of sonography to visualize and identify the ROI of the hip neck provides the opportunity to identify occult fractures. Such fractures are often hard to diagnose and missed by practitioners due to their atypical clinical and radiographic manifestation [15]. Fractures of the proximal femur are a common type of fragility fractures [16]. The ultrasound image of occult fractures may not always appear clearly as a break of the bone cortex, but also as an interference or abnormality of the surrounding tissue. The scan of the other hip is useful as a comparison. In addition, the joint could be examined for effusion. [17] The gold standard for diagnosis when plain radiographs and CT yield negative results is the MRI exam [18]. This method is costly and not highly available. On the other hand, bone density measurements such as REMS are carried out regularly and performed by wide variety of specialists.

**Figure 4:**
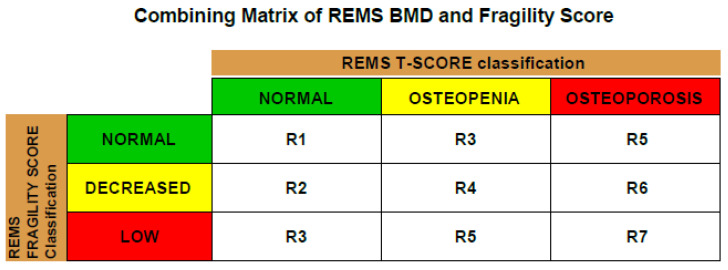
Combining matrix of REMS BMD against fragility score (each diagnosis has 3 classes of fragility score). Fragility score is a dimensionless parameter that allows the estimation of skeletal fragility using the ultrasound scan performed with REMS, and it can vary from 0 to 100. The physician can identify the risk class corresponding to the current patient by combining the measured REMS T-score and FS values using the combining matrix. The fragility score is an accurate estimation of the risk of osteoporotic (fragility) fractures at 5 years [19]. These include fractures of the proximal femur, the vertebrae, the proximal humerus and the distal radius caused by low-energy or minor trauma, such as a fall from standing height [20]. The potential to simultaneously diagnose osteoporosis and fragility fractures proves this approach to be effective. This allows preventive measures to be taken to avoid the secondary displacement of the fracture, immobilization and complications following the future treatment [21]. Furthermore, the adequate treatment of the primary disease osteoporosis can be initiated to prevent further fractures.

**Figure 5:**
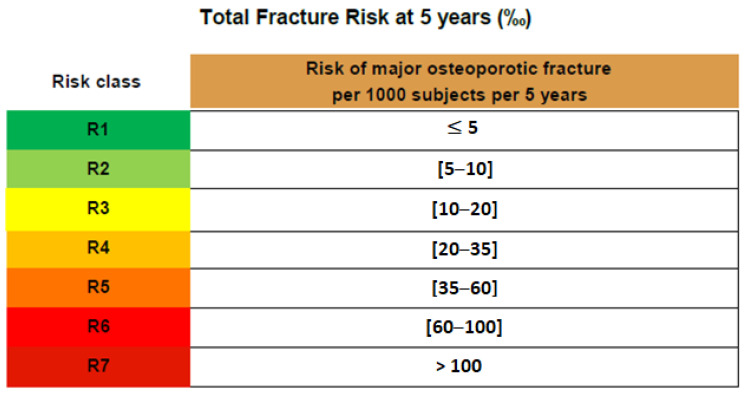
REMS definition of risk classes according to fragility score. Risk class can range from R1 to R7. A higher risk class corresponds to a higher fracture risk. Once the risk class is identified for the patient through the matrix described above, it is possible to quantify the patient-associated fracture risk range expressed in terms of ‰ per 5 years. In our case, the patient was classified into class R7 for hip and class R6 for spine, meaning that the risk of osteoporotic fracture of the hip at 5 years was much higher than that of the spine. BMD and T-score alone are not sufficient measurements when it comes to the diagnosis and treatment of osteoporosis. The microarchitecture of the bone tissue plays an important role too. The REMS technology provides a combining matrix of the REMS BMD against the fragility score in order to obtain a full picture of the condition and enhance decision making. Occult fractures of the femoral neck are hard to diagnose and require numerous radiologic exams. Recent studies show the importance of ultrasound methods. The REMS method for measuring bone density using ultrasound allows the simultaneous diagnosis of osteoporosis and estimation of the fracture risk. It is accessible to large number of specialists, and the ultrasound analysis offers the opportunity to discover occult femoral neck fractures, especially in cases where patients are bedridden and other diagnostic methods are hard to perform. In addition to the sonographic scan, the possibility to diagnose osteoporosis with the REMS method is helpful for initiating osteoporosis treatment.

## Data Availability

The data supporting the conclusions of this article are included within the article.

## List of Abbreviations

BMD	bone mineral density
CT	computed tomography
DEXA	dual-X-ray-absorptiometry
MRI	magnetic resonance imaging
REMS	radiofrequency echographic multi-spectrometry
RF	radio frequency
ROI	region of interest
